# Methionine, Homocysteine, and Methylation Levels Predict Cognitive Decline in Alzheimer's Disease

**DOI:** 10.1002/cns.70954

**Published:** 2026-05-27

**Authors:** Xiaoyu Zhang, Ai Guan, Wanlin Li, Zhenbo Zhao, Liufeng Tang, Xiaoguang Yao, Xiaoyun Liu, Gang Wang, Hualong Wang

**Affiliations:** ^1^ Department of Neurology, Renji Hospital Shanghai Jiaotong University School of Medicine Shanghai China; ^2^ Department of Neurology, The First Hospital of Hebei Medical University Brain Aging and Cognitive Neuroscience Laboratory of Hebei Province Shijiazhuang China; ^3^ Hebei Key Laboratory of Integrative Medicine on Liver‐Kidney Patterns, College of Integrative Medicine, Institute of Integrative Medicine Hebei University of Chinese Medicine Shijiazhuang China

**Keywords:** Alzheimer's disease, homocysteine, methionine, methylation, white matter hyperintensities

## Abstract

**Background:**

Alzheimer's disease (AD), the most common cause of dementia, involves metabolic factors such as homocysteine, methionine, and DNA methylation in its pathogenesis. However, the precise interactions among these factors and their impact on cognitive function in AD require further elucidation.

**Methods:**

A total of 191 AD patients and 200 cognitively normal (CN) individuals were selected from the Alzheimer's Disease Neuroimaging Initiative (ADNI) database. Demographic, clinical, and metabolic data, including MMSE scores and white matter hyperintensity (WMH) volumes, were collected. Longitudinal cognitive changes over 1 year were also evaluated in 104 AD patients.

**Results:**

Compared to CN subjects, AD patients exhibited significantly lower MMSE scores, hippocampal volume, total brain volume, methionine levels, and methylation levels, alongside higher homocysteine levels and WMH volumes. Logistic regression and correlation analyses revealed that homocysteine, methionine, and methylation levels were significantly associated with cognitive function in AD patients but not in CN subjects. Elevated homocysteine levels in AD patients correlated with lower MMSE scores, higher WMH volumes, lower vitamin B12 levels, and older age. Similarly, low methionine and methylation levels were associated with more severe cognitive impairment and brain atrophy. Longitudinal analyses showed that AD patients with low methionine, high homocysteine, or low methylation levels experienced significantly greater cognitive decline over 1 year compared to those with more favorable biomarker profiles.

**Conclusions:**

Our findings highlight the significant associations between homocysteine, methionine, and methylation levels and cognitive decline in AD patients. These results suggest that metabolic and epigenetic alterations may contribute to AD progression and could serve as potential biomarkers or therapeutic targets in AD.

## Graphical abstract

Elevated homocysteine and reduced methionine/methylation levels predict accelerated cognitive decline and white matter damage over one year in AD patients. This metabolic imbalance reveals a modifiable pathway linked to vitamin B12 deficiency. Targeting one‐carbon metabolism may provide novel therapeutic strategies to delay neurodegeneration.

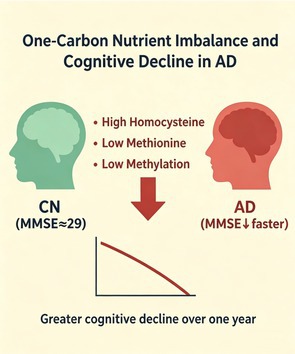

## 1 Introduction

Alzheimer's disease (AD) is a chronic and progressive neurodegenerative disorder that is the most common cause of dementia [1], primarily characterized by cognitive impairment and psychiatric or behavioral disturbances [2]. Despite extensive research into its pathophysiology, the precise mechanisms underlying cognitive decline in AD remain incompletely understood. Emerging evidence suggests that metabolic factors, including methionine, homocysteine, and DNA methylation, may play critical roles in the development and progression of AD [3, 4].

Homocysteine, a sulfur‐containing amino acid, has been implicated in neurodegeneration due to its potential to induce vascular damage and promote oxidative stress [5]. Elevated homocysteine levels have been associated with increased risk of cognitive decline and dementia [6]. Recent studies have found that homocysteine levels in AD patients are higher than those in healthy controls, and this increase may have occurred decades before the onset of the disease [7]. Conversely, methionine, a precursor of S‐adenosylmethionine (SAM), is involved in one‐carbon metabolism and methylation reactions critical for maintaining neuronal integrity [8]. Altered methylation levels can influence gene expression and epigenetic regulation, potentially contributing to the pathogenesis of AD [9]. Vitamin B12, a crucial cofactor in the methionine cycle, plays a vital role in maintaining low homocysteine levels and supporting methylation reactions [10]. Deficiency of vitamin B12 can lead to elevated homocysteine and impaired methylation capacity, potentially accelerating cognitive decline in AD [11, 12].

Although the association between metabolites of the vitamin B12 metabolic pathway and AD has been preliminarily confirmed, existing findings remain inconsistent. Moreover, previous studies have mainly focused on isolated analysis of a single metabolic marker (such as homocysteine), while integrated analysis of multiple key metabolites remains insufficient. Additionally, most of these studies are based on cross‐sectional designs, making it difficult to reveal the dynamic relationship between metabolic factors and cognitive decline.

To address these gaps, this study, based on the Alzheimer's Disease Neuroimaging Initiative (ADNI), aimed to observe the distribution characteristics of metabolites related to the vitamin B12 metabolic pathway in populations with different cognitive stages, systematically explore their specific associations with the cognitive function of AD patients, and use longitudinal follow‐up data to evaluate the impact of these metabolic factors on the cognitive decline of AD patients. These findings may provide new insights into the role of these biomarkers in AD progression and guide potential intervention strategies.

## 2 Methods

### 2.1 Data Collection

This research is based on data from the Alzheimer's Disease Neuroimaging Initiative (ADNI) database (adni.loni.usc.edu). The ADNI was launched in 2003 as a public‐private partnership, led by Principal Investigator Michael W. Weiner, MD. ADNI is a longitudinal, multi‐center, observational study. The primary goal of ADNI has been to test whether serial magnetic resonance imaging (MRI), positron emission tomography (PET), other biological markers, and clinical and neuropsychological assessment can be combined to measure the progression of mild cognitive impairment (MCI) and early AD. All involved institutions have given their approval for accessing the ADNI database, with signed consent forms from participants at each location. This research project has been consented to by the initiative's responsible parties.

### 2.2 Study Participants

A total of 391 subjects were included in this study, with 191 in the AD group and 200 in the CN group. General inclusion criteria for the different patient groups were as follows: AD subjects: CDR > 0.5, meets NIA‐AA criteria for AD. Exclusion criteria include neurologic conditions other than AD that could affect cognition, such as Parkinson's disease and Vascular Dementia; excluded participants taking vitamin B12 supplements or multivitamins. Inclusion and exclusion criteria are detailed in the ADNI protocol [13]. CN subjects: MMSE scores between 24 and 30 (inclusive), a CDR of 0, cognitively normal, based on an absence of significant impairment in cognitive functions or activities of daily living; excluded participants taking vitamin B12 supplements or multivitamins. The patients were further categorized into two groups (lower and higher levels) according to the median concentrations of methionine, methylation levels, and homocysteine.

### 2.3 Biochemical Analyses

Blood plasma samples were collected before breakfast on the morning of the baseline MRI scans, after an overnight fast in order to avoid inaccuracies due to recent consumption of certain foods. Total homocysteine levels were measured in a sample of blood plasma taken from each subject using a validated enzyme immunoassay [14]. Vitamin B12 levels were assayed using a Siemens ADVIA Centaur XP autoanalyzer immunoassay (ACI) conducted by Quest Diagnostics [15]. Methionine levels were measured using an ultra‐performance liquid chromatography–tandem mass spectrometry (UPLC‐MS/MS) assay conducted at the University of Hawaii Cancer Center. In this study, the ratio of methionine to homocysteine (Met/Hcy) was used as a surrogate indicator to reflect the overall methylation potential. A lower ratio suggests an obstruction in the methylation cycle.

### 2.4 Neuropsychological Testing

Cognitive testing was available for both ADNI and AIBL. ADNI utilizes an extensive battery of assessments to examine cognitive functioning with particular emphasis on domains relevant to AD. All subjects underwent clinical and neuropsychological assessment at the time of scan acquisition. The MMSE is a fully structured screening instrument frequently used for AD drug studies. The scale evaluates orientation to place, orientation to time, registration (immediate repetition of three words), attention and concentration (serially subtracting seven beginning with 100), recall (recalling the previously repeated three words), language (naming, repetition, reading, writing, comprehension), and visual construction (copy two intersecting pentagons). The MMSE is scored as the number of correctly completed items, with lower scores indicative of poorer performance and greater cognitive impairment. The total score ranges from 0 to 30 (perfect performance). Permissible scores for each category of subjects are listed in the inclusion criteria.

### 2.5 WMH Volume Measurement

The details of this method are published [16]. This approach utilizes run‐time PD‐, T1‐, and T2‐weighted structural magnetic resonance (MR) images, along with labeled training examples derived from FLAIR‐based WMH detections. The method involves learning probabilistic models of WMH spatial distribution and neighborhood dependencies, which are then incorporated into a Markov Random Field (MRF) framework to infer WMH positions in novel test images. All MR scans were preprocessed by rigidly coregistering T1, T2, PD, and FLAIR images using cross‐correlation, followed by manual segmentation of nonbrain tissues and nonlinear alignment of the skull‐stripped T1‐weighted image to a minimum deformation template (MDT). The detection employs a Bayesian MRF approach, maximizing the posterior probability of binary labels for each voxel based on the image intensity data, influenced by spatial and contextual priors. The method was trained using FLAIR‐based ground‐truth WMH detections and validated on diverse datasets, demonstrating robust performance with at least 60 training images and achieving high interclass correlation coefficients (ICC) when classifying images from different populations and scanners.

### 2.6 Statistical Analysis

The statistical analyses were conducted using the SPSS 26.0 statistical software. All statistical tests were two‐tailed, and *p* < 0.05 was considered statistically significant. Continuous variables were summarized as mean ± standard deviation (SD) or median (M, P25, P75). Categorical data were presented as percentages (%). For continuous variables that followed a normal distribution, comparisons between two independent groups were performed using the independent samples *t*‐test. For continuous variables that did not follow a normal distribution, comparisons between two independent groups were conducted using the nonparametric Mann–Whitney *U* test. For categorical data, comparisons between groups were made using the chi‐square test. Correlation analyses were conducted using Pearson's correlation coefficient for normally distributed variables and Spearman's rank correlation coefficient for non‐normally distributed variables. Binary logistic regression analysis was employed to examine the relationships between methionine, methylation levels, homocysteine, and AD.

## 3 Results

### 3.1 Demographic and Clinical Characteristics

The demographic and clinical characteristics of the 200 CN and 191 AD subjects are presented in Table 1. There were no significant differences between the CN and AD groups in terms of age (CN: 76.92 ± 5.6; AD: 76.62 ± 7.2; *p* = 0.624), sex (male ratio in CN: 51%, AD: 58.6%; *p* = 0.129), cardiovascular disease (CN: 66.5%, AD: 68.1%; *p* = 0.742), diabetes mellitus (CN: 41%, AD: 39.3%; *p* = 0.727), smoking (CN: 36.5%, AD: 38.7%; *p* = 0.647), and vitamin B12 levels (CN: 509.31 ± 342.8; AD: 509.35 ± 279.3; *p* = 0.999). The AD group exhibited significantly lower MMSE scores (CN: 29.08 ± 1.1; AD: 22.35 ± 3.9; *p* = 0.000), hippocampal volume (CN: 7290.9 ± 826.8; AD: 5694.2 ± 1114.3; *p* = 0.000), total brain volume (CN: 1054.8 ± 103.9; AD: 1000.6 ± 119.5; *p* = 0.000), methionine levels (CN: 26.38 ± 5.4; AD: 24.98 ± 3.9; *p* = 0.003), and methylation levels (CN: 2.71 ± 0.8; AD: 2.41 ± 0.7; *p* = 0.000). The AD group had significantly higher homocysteine (CN: 10.16 ± 2.3; AD: 11.06 ± 2.9; *p* = 0.001) and WMH volume (CN: 0.249 (0.082, 0.624); AD: 0.639 (0.211, 1.430); *p* = 0.000). Additionally, the AD group had a higher proportion of APOE4 carriers (CN: 26.5%, AD: 71.2%; *p* = 0.000) and fewer years of education (CN: 16.11 ± 2.6; AD: 15.28 ± 2.8; *p* = 0.003).

**TABLE 1:** Demographic and clinical characteristics of the subjects.

Characteristic	CN *N* = 200	AD *N* = 191	*p*
Male *n* (%)	102 (51.0%)	112 (58.6%)	0.129
Smoking, *n* (%)	73 (36.5%)	74 (38.7%)	0.647
Diabetes mellitus, *n* (%)	82 (41%)	75 (39.3%)	0.727
Cardiovascular disease, *n* (%)	133 (66.5%)	130 (68.1%)	0.742
APOE4 allele, *n* (%)	53 (26.5%)	136 (71.2%)	0.000
MMSE	29.08 ± 1.1	22.35 ± 3.9	0.000
Age (years)	76.92 ± 5.6	76.60 ± 7.2	0.624
Education (years)	16.11 ± 2.6	15.28 ± 2.8	0.003
Methionine (μmol/L)	26.38 ± 5.4	24.98 ± 3.9	0.003
Homocysteine (μM)	10.16 ± 2.3	11.06 ± 2.9	0.001
Methylation (Met/Hcy)	2.71 ± 0.8	2.41 ± 0.7	0.000
Vitamin B12 (pg/mL)	509.31 ± 342.8	509.35 ± 279.3	0.999
WMH volume (mL)	0.249 (0.082, 0.624)	0.639 (0.211, 1.403)	0.000
Hippocampal volume (mm^3^)	7290.9 ± 826.8	5694.2 ± 1114.3	0.000
Total brain volume (mL)	1054.8 ± 103.9	1000.6 ± 119.5	0.000

### 3.2 Association of Cognitive Function With Homocysteine, Methionine, and Methylation Levels in AD Patients

**FIGURE 1:**
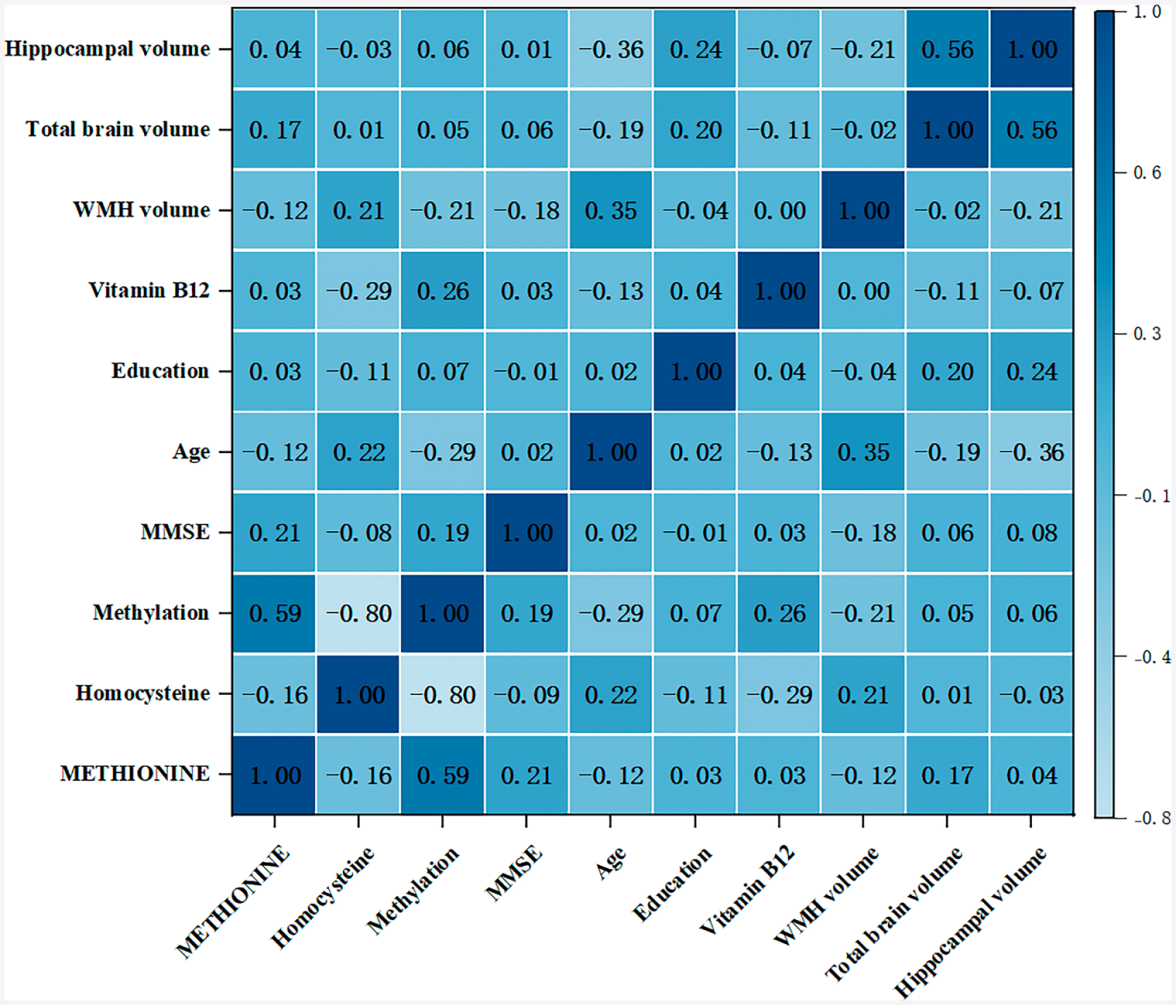
Correlation coefficients among key metabolic and clinical indicators in AD.

After adjusting for confounding factors including age, education, binary logistic regression analysis revealed that methionine levels, methylation levels, and homocysteine levels were significantly associated with AD (Table S1, methionine: 0.026; methylation: 0.032; homocysteine: 0.008). Pearson and Spearman correlation analyses indicated that homocysteine levels were positively correlated with WMH volume (*r* = 0.211, *p* = 0.004), age (*r* = 0.219, *p* = 0.002), and negatively correlated with serum vitamin B12 levels (*r* = −0.293, *p* = 0.000). Additionally, WMH volume was negatively correlated with MMSE scores (*r* = −0.176, *p* = 0.015) (Figure 1). However, no significant correlations were found in CN patients (Figure S1, homocysteine vs. WMH: *p* = 0.452; WMH vs. MMSE: *p* = 0.474). Compared to AD patients with lower homocysteine levels, those in the high homocysteine group had lower MMSE scores, were older, had lower vitamin B12 levels, and had higher WMH volume (Table 2). In terms of methionine levels, the low methionine group showed lower MMSE scores, a higher proportion of females, older age, and more severe brain atrophy. Similarly, the low methylation group was characterized by lower MMSE scores, older age, reduced vitamin B12 levels, and increased WMH volume. These associations were not observed in CN subjects (Table S2, lower homocysteine WMH vs. higher homocysteine WMH: *p* = 0.602; lower methionine brain volume vs. higher brain volume: *p* = 0.292; lower methylation MMSE vs. higher methylation MMSE: *p* = 0.792). Further analysis indicated that vitamin B12 was independently associated with both homocysteine and methylation levels (Tables S3 and S4, homocysteine group: *p* = 0.000; methylation group: *p* = 0.004).

**TABLE 2:** Analysis of clinical characteristics in AD patients based on homocysteine, methionine and methylation levels.

Variables	Low homocysteine	High homocysteine	*p*	Low methionine	High methionine	*p*	Low methylation	High methylation	*p*
Female, *n* (%)	39 (41.1%)	40 (41.7%)	0.931	50 (51.5%)	29 (30.9%)	0.004	44 (44.9%)	35 (37.6%)	0.308
Smoking, *n* (%)	39 (41.1%)	35 (36.5%)	0.515	38 (39.2%)	36 (38.3%)	0.901	33 (33.7%)	41 (44.1%)	0.140
Diabetes mellitus, *n* (%)	38 (40.0%)	37 (38.5%)	0.837	36 (37.1%)	39 (41.5%)	0.536	38 (38.8%)	37 (39.8%)	0.886
Cardiovascular disease	62 (65.3%)	68 (70.8%)	0.409	66 (68.0%)	64 (68.1%)	0.995	71 (72.4%)	59 (63.4%)	0.182
APOE4 allele, *n* (%)	76 (80.0%)	60 (62.5%)	0.008	69 (71.1%)	67 (71.3%)	0.983	64 (65.3%)	72 (77.4%)	0.065
MMSE	22.9 ± 3.2	21.8 ± 4.4	0.038	21.6 ± 3.8	23.1 ± 3.9	0.007	21.5 ± 4.4	23.2 ± 3.0	0.002
Age (years)	75.0 ± 7.4	78.2 ± 6.7	0.002	78.0 ± 6.9	75.1 ± 7.3	0.005	78.1 ± 6.7	75.0 ± 7.4	0.002
Education (years)	15.4 ± 2.7	15.2 ± 3.0	0.538	15.3 ± 2.9	15.2 ± 2.8	0.777	15.2 ± 3.0	15.3 ± 2.7	0.811
Vitamin B12 (pg/mL)	597.6 ± 324.5	423.1 ± 190.1	0.000	487.5 ± 204.9	538.9 ± 337.6	0.164	445.5 ± 194.7	576.1 ± 334.7	0.002
WMH volume (mL)	0.437 (0.174, 1.043)	0.838 (0.251, 1.986)	0.005	0.806 (0.287, 1.760)	0.424 (0.136, 1.115)	0.010	0.816 (0.287, 2.056)	0.419 (0.151, 1.011)	0.002
Hippocampal volume (mm^3^)	5627.7 ± 1070.4	5765.3 ± 1161.2	0.407	5604.8 ± 1110.8	5798.8 ± 1116.4	0.264	5725.2 ± 1164.5	5662.5 ± 1066.0	0.705
Total brain volume (mL)	999.4 ± 115.1	1001.7 ± 124.0	0.894	980.6 ± 117.3	1021.3 ± 118.8	0.019	997.0 ± 122.8	1004.5 ± 116.3	0.671
Methionine (μmol/L)	25.4 ± 3.7	24.6 ± 4.0	0.185						
Homocysteine (μM)				11.4 ± 3.3	10.7 ± 2.5	0.149			

### 3.3 Longitudinal Effects of Methionine, Homocysteine, and Methylation Levels on Cognitive Decline in AD Patients

To further investigate the dynamic effects of methionine, homocysteine, and methylation levels on cognitive function, we collected data from 104 AD patients, including basic characteristics, baseline MMSE scores, MMSE scores after 1 year, and the change in MMSE scores over the year (Table 3). The results showed that the baseline MMSE scores (Table 3, methionine group *p* = 0.003; homocysteine group *p* = 0.037; methylation group *p* = 0.001), MMSE scores after 1 year (Table 3, methionine group *p* = 0.000; homocysteine group *p* = 0.001; methylation group *p* = 0.000), and the change in MMSE scores (Table 3, methionine group *p* = 0.036; homocysteine group *p* = 0.011; methylation group *p* = 0.011) all declined significantly more in the low methionine group, high homocysteine group, and low methylation group, respectively, compared with the high methionine, low homocysteine, and high methylation groups.

**TABLE 3:** Baseline profile and longitudinal changes in MMSE scores over 1 year.

Variables	Low homocysteine	High homocysteine	*p*	Low methionine	High methionine	*p*	Low methylation	High methylation	*p*
Female, *n* (%)	20 (38.5%)	25 (49.0%)	0.280	32 (60.4%)	13 (26.0%)	0.000	28 (54.9%)	17 (32.7%)	0.023
Smoking, *n* (%)	22 (42.3%)	14 (27.5%)	0.114	16 (30.2%)	20 (40.0%)	0.297	11 (21.6%)	25 (48.1%)	0.005
Diabetes mellitus, *n* (%)	21 (40.4%)	21 (41.2%)	0.935	20 (37.7%)	22 (44.0%)	0.518	22 (43.1%)	20 (38.5%)	0.629
Cardiovascular disease	34 (65.4%)	39 (76.5%)	0.216	36 (67.9%)	37 (74.0%)	0.498	39 (76.8%)	34 (65.4%)	0.216
APOE4 allele, *n* (%)	40 (76.9%)	34 (66.7%)	0.247	38 (71.7%)	36 (72.0%)	0.973	34 (66.7%)	40 (76.9%)	0.247
MMSE	23.7 ± 2.4	22.5 ± 3.4	0.037	22.3 ± 2.8	24.0 ± 3.0	0.003	22.1 ± 3.4	24.1 ± 2.1	0.001
Age (years)	75.2 ± 7.5	78.7 ± 6.3	0.011	78.1 ± 7.0	75.7 ± 7.0	0.083	78.5 ± 6.5	75.4 ± 7.4	0.029
Education (years)	15.5 ± 3.0	14.6 ± 3.1	0.140	15.0 ± 2.9	15.1 ± 3.2	0.945	14.5 ± 2.9	15.6 ± 3.1	0.083
Vitamin B12 (pg/mL)	591.1 ± 342.7	395.3 ± 149.5	0.000	490.5 ± 184.9	502.5 ± 365.1	0.837	416.2 ± 158.1	577.9 ± 353.6	0.005
WMH volume (mL)	0.393 (0.122, 1.195)	0.741 (0.267, 1.429)	0.113	0.777 (0.275, 1.356)	0.387 (0.110, 1.080)	0.093	0.750 (0.267, 1.429)	0.414 (0.105, 1.046)	0.066
Hippocampal volume (mm^3^)	5753.6 ± 1091.3	5631.2 ± 1089.2	0.582	5434.8 ± 1095.3	5996.9 ± 1005.7	0.010	5509.8 ± 1076.8	5870.2 ± 1076.8	0.103
Total brain volume (mL)	1005.3 ± 104.4	974.1 ± 116.2	0.156	962.8 ± 113.3	1017.8 ± 102.4	0.012	968.4 ± 117.4	1011.0 ± 100.9	0.052
MMSE (1 year follow‐up)	21.9 ± 3.7	19.1 ± 4.8	0.001	19.1 ± 3.9	22.1 ± 4.6	0.000	18.7 ± 4.8	22.3 ± 3.5	0.000
ΔMMSE	1.77 ± 3.3	3.4 ± 2.9	0.011	3.2 ± 3.3	1.9 ± 3.0	0.036	3.4 ± 3.1	1.8 ± 3.0	0.011
Methionine (μmol/L)	25.2 ± 3.8	25.1 ± 3.8	0.882						
Homocysteine (μM)				11.0 ± 3.1	11.2 ± 2.8	0.773			

## 4 Discussion

In this study, we investigated the associations of methionine, homocysteine, and methylation levels with cognitive function in Alzheimer's disease (AD) patients compared to cognitively normal (CN) individuals and further explored their impact on longitudinal cognitive decline in AD. Our findings provide additional evidence that disruptions in these metabolic pathways may contribute to the pathophysiology and progression of AD.

Consistent with previous studies, we found that homocysteine levels were significantly elevated in AD patients, while methionine and methylation levels were significantly reduced compared to CN subjects [3]. Elevated homocysteine has been proposed as a neurotoxic factor, promoting oxidative stress, endothelial dysfunction, and blood–brain barrier disruption, all of which can accelerate neurodegenerative processes [17, 18, 19]. The positive correlation we observed between homocysteine levels and white matter hyperintensity (WMH) volume further supports the link between homocysteine and cerebrovascular damage in AD. Moreover, the negative correlation between WMH volume and MMSE scores underscores the clinical relevance of homocysteine‐mediated vascular injury and cognitive impairment.

Most previous studies have focused on the relationship between vitamin B12, homocysteine, and AD. In contrast, few studies have explored the relationship between methionine levels, methylation levels, and AD. Methionine, as a precursor of S‐adenosylmethionine (SAM), plays a central role in methyl group donation for DNA methylation and epigenetic regulation [9, 10, 20]. Our data found that in AD patients, lower methionine levels, lower methylation levels, and higher homocysteine levels were associated with poor cognitive function. The observed reductions in methionine and methylation levels in AD patients may compromise DNA methylation patterns, potentially affecting the expression of genes critical for neuronal function and plasticity [21, 22]. Notably, the relationship between methionine and AD remains controversial. Some animal studies have shown that exogenous methionine supplementation in adult mice can exacerbate neuroinflammation and impair cognitive function [23]. In contrast, a longitudinal, population‐based study reported that higher methionine and methylation levels were associated with a reduced risk of dementia [3]. These findings highlight the complexity of methionine metabolism in the context of neurodegeneration and underscore the need for further research to clarify its role in AD pathogenesis.

Furthermore, our longitudinal analysis revealed that low methionine, high homocysteine, and low methylation levels at baseline were each independently associated with more pronounced cognitive decline over a 1‐year period in AD patients. This suggests that these metabolic factors may not only contribute to the onset of cognitive impairment but also accelerate disease progression. Importantly, we found that vitamin B12 levels were inversely correlated with homocysteine and positively correlated with methylation levels, supporting the critical role of vitamin B12 as a cofactor in the methionine cycle and methylation reactions [24]. Vitamin B12 deficiency may exacerbate homocysteine accumulation and compromise methylation capacity, thereby accelerating cognitive decline in AD [25]. Interestingly, these associations were not significant in CN individuals, suggesting that disruptions in methionine metabolism and methylation may become clinically relevant only in the context of neurodegeneration or increased vulnerability to AD pathology. This highlights the potential of methionine, homocysteine, and methylation as biomarkers of disease progression in AD, rather than markers of normal aging.

However, in our study, no direct correlation between vitamin B12 and AD patient cognition was found. This finding is inconsistent with some previous studies reporting that vitamin B12 deficiency is associated with increased risk of cognitive decline [11, 12]. Several factors may explain this discrepancy. First, serum vitamin B12 levels may not reflect its bioactive status in tissues. The serum vitamin B12 level measured actually represents the sum of its various bound forms and does not indicate the actual tissue uptake and utilization efficiency [26]. Second, the mean vitamin B12 levels in both AD patients and cognitively normal individuals in this study exceeded 500 pg/mL, which is above the clinical deficiency threshold. In a nutritionally adequate population, individual variations in vitamin B12 levels may be insufficient to produce detectable effects on cognitive function [27]. Third, longitudinal studies have shown that the effects of vitamin B12 on brain structure require a longer cumulative period, and the direct association with AD risk may not be readily apparent through short‐term changes in serum levels [28]. Therefore, the role of vitamin B12 in AD may be primarily indirect, mediated through its regulation of homocysteine and methylation metabolism, rather than through direct effects on cognitive function.

Our study has several limitations. First, the lack of a direct association between vitamin B12 and cognitive function may partly reflect the limited sample size and relatively short follow‐up period of our longitudinal cohort, which may have been insufficient to detect subtle longitudinal effects. Second, although we adjusted for key confounders, residual confounding cannot be fully excluded in an observational study. Third, we measured global methylation levels rather than site‐specific DNA methylation changes, which may limit insights into specific gene regulation. Finally, the cross‐sectional design of most of our data precludes definitive conclusions about causality.

## 5 Conclusion

In conclusion, our study demonstrates that elevated homocysteine and reduced methionine and methylation levels are associated with worse cognitive performance and accelerated cognitive decline in AD patients. These findings underscore the importance of metabolic factors in AD pathophysiology and highlight potential avenues for therapeutic intervention, including homocysteine‐lowering strategies and methylation support through dietary or pharmacological approaches. Future longitudinal and mechanistic studies are needed to clarify the precise roles of these metabolic alterations in AD progression and to evaluate their potential as therapeutic targets.

## Author Contributions

**Xiaoyu Zhang:** conceptualization, data curation, formal analysis, writing – original draft. **Ai Guan:** conceptualization, methodology, writing – original draft. **Wanlin Li:** data curation, formal analysis, writing – review and editing. **Zhenbo Zhao:** data curation, writing – review and editing. **Liufeng Tang:** data curation, writing – review and editing. **Xiaoguang Yao:** data curation, writing – review and editing. **Xiaoyun Liu:** methodology, writing – review and editing. **Gang Wang:** conceptualization, methodology, supervision, writing – review and editing. **Hualong Wang:** funding acquisition, conceptualization, methodology, writing – review and editing. All authors discussed the results and reviewed the manuscript.

## Funding

This work was supported by National Natural Science Foundation of China (82371416), Natural Science Foundation of Hebei Province (H2024206494 and H2024423038), and Science and Technology Research Project for Higher Education Institutions in Hebei Province (ZD2022146), Government‐funded Outstanding Clinical Medical Talent Training Program (ZF2025047).

## Ethics Statement

ADNI was approved by the institutional review boards of all participating centers, and written informed consent was obtained from all participants or authorized representatives according to the 1975 Declaration of Helsinki.

## Conflicts of Interest

The authors declare no conflicts of interest.

## Supporting information


**Figure S1:** Correlation coefficients among key metabolic and clinical indicators in CN.

**Table S1:** Independent risk factors for AD.
**Table S2:** Analysis of clinical characteristics in NC subjects based on homocysteine, methionine and methylation levels.
**Table S3:** Independent factors associated with homocysteine levels in AD patients.
**Table S4:** Independent factors associated with methylation levels in AD patients.

## Data Availability

Data used in the preparation of this article were obtained from the Alzheimer's Disease Neuroimaging Initiative (ADNI) database (adni.loni.usc.edu). The investigators within the ADNI contributed to the design and implementation of ADNI and/or provided data but did not participate in analysis or writing of this report. A complete listing of ADNI investigators can be found at: http://adni.loni.usc.edu/wp‐content/uploads/how_to_apply/ADNI_Acknowledgement_List.pdf.
