# Supplementary material for: Methionine, Homocysteine, and Methylation Levels Predict Cognitive Decline in Alzheimer's Disease
Source: CNS Neurosci Ther. 2026 May 27;32(6):e70954. doi: 10.1002/cns.70954 (PMC13240123; doi:10.1002/cns.70954)
Supplement: Supplementary file 1 — Figure S1: Correlation coefficients among key metabolic and clinical indicators in CN. [file CNS-32-e70954-s001.docx]

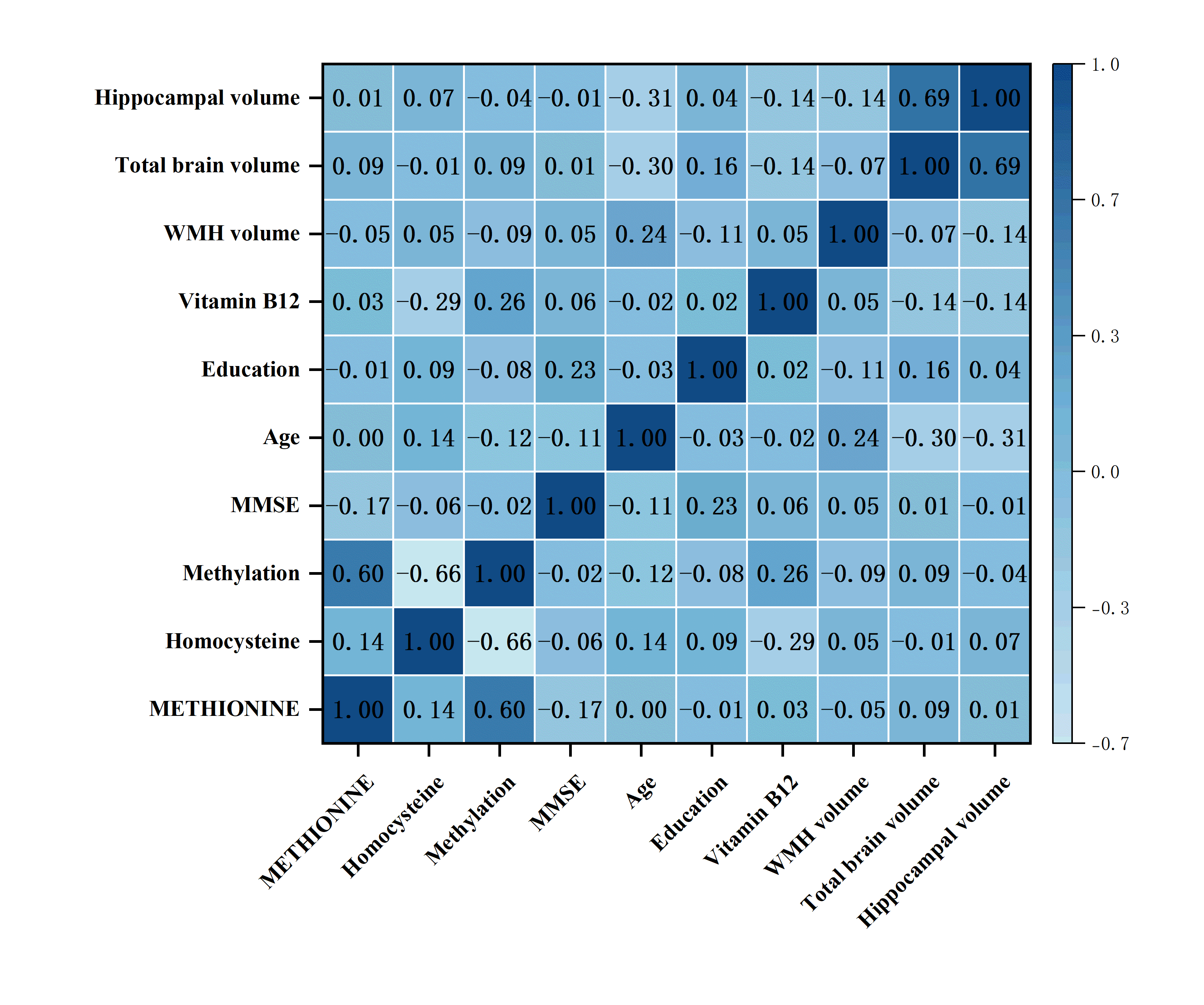


Supplemental Figure 1. Correlation coefficients among key metabolic and clinical indicators in CN
