# Supplementary material for: Methionine, Homocysteine, and Methylation Levels Predict Cognitive Decline in Alzheimer's Disease
Source: CNS Neurosci Ther. 2026 May 27;32(6):e70954. doi: 10.1002/cns.70954 (PMC13240123; doi:10.1002/cns.70954)
Supplement: Supplementary file 2 — Table S1: Independent risk factors for AD. Table S2: Analysis of clinical characteristics in NC subjects based on homocysteine, methionine and methylation levels. Table S3: Independent factors associated with homocysteine levels in AD patients. Table S4: Independent factors associated with methylation levels in AD patients. [file CNS-32-e70954-s002.docx]

Supplemental Table 1. Independent risk factors for AD

| **Variable** | **OR** | ***P*** |
| --- | --- | --- |
| Methionine | 0.70（0.508, 0.958） | 0.026 |
| Homocysteine | 2.53（1.279, 5.020） | 0.008 |
| Methylation | 25.55（1.314, 421.838） | 0.032 |
| Hippocampal volume | 0.998（0.997, 0.999） | 0.000 |
| MMSE | 0.158（0.084, 0.298） | 0.000 |

Supplemental Table 2: Analysis of clinical characteristics in NC subjects based on homocysteine, methionine and methylation levels

| **Variables** | **low Methionine** | **high Methionine** | ***P*** | **low Homocysteine** | **high Homocysteine** | ***P*** | **Low Methylation** | **High Methylation** | ***P*** |
| --- | --- | --- | --- | --- | --- | --- | --- | --- | --- |
| Male n (%) | 38（38%） | 64（64%） | 0.000 | 38（38.4%） | 64（63.4%） | 0.000 | 50（50.5%） | 48（47.5%） | 0.673 |
| Smoking, n (%) | 33（33%） | 40（40%） | 0.304 | 25（25.3%） | 48（47.5%） | 0.001 | 38（38.4%） | 35（34.7%） | 0.584 |
| Diabetes mellitus, n (%) | 43（43%） | 39（39%） | 0.565 | 31（31.3%） | 51（50.5%） | 0.006 | 50（50.5%） | 32（31.7%） | 0.007 |
| Cardiovascular disease, | 64（64%） | 69（69%） | 0.454 | 59（59.6%） | 74（73.3%） | 0.041 | 72（72.7%） | 61（60.4%） | 0.065 |
| APOE4 allele, n (%) | 25（25%） | 28（28%） | 0.631 | 23（23.2%） | 30（29.7%） | 0.300 | 26（26.3%） | 27（26.7%） | 0.940 |
| MMSE | 29.3±0.9 | 28.9±1.2 | 0.007 | 29.2±1.0 | 28.9±1.3 | 0.040 | 29.1±1.1 | 29.1±1.1 | 0.792 |
| Age (years) | 76.9±6.1 | 76.9±5.2 | 0.940 | 76.6±5.4 | 77.2±6.0 | 0.423 | 77.8±6.3 | 76.1±4.9 | 0.048 |
| Education (years) | 16.0±2.7 | 16.2±2.6 | 0.646 | 15.8±2.8 | 16.4±2.4 | 0.089 | 16.3±2.5 | 15.9±2.7 | 0.265 |
| Vitamin B12(pg/mL) | 481.8±297.3 | 536.25±381.7 | 0.265 | 606.6±405.4 | 414.0±232.8 | 0.000 | 446.9±311.0 | 569.2±362.2 | 0.011 |
| WMH volume (ml) | 0.262(0.082,0.771) | 0.197(0.080,0.476) | 0.274 | 0.240(0.076,0.639) | 0.257(0.113,0.608) | 0.602 | 0.274(0.117,0.623) | 0.197(0.707,0.624) | 0.177 |
| Hippocampal volume(mm3) | 7281.4±853.9 | 7300.3±803.6 | 0.877 | 7244.1±823.8 | 7333.3±831.5 | 0.466 | 7293.7±853.3 | 7287.9±802.6 | 0.962 |
| Total brain volume(ml) | 1047.1±110.0 | 1062.6±97.4 | 0.292 | 1056.2±105.0 | 1053.5±103.5 | 0.851 | 1045.8±110.9 | 1063.7±96.3 | 0.223 |
| Methionine(µmol/L) |  |  |  | 25.7±5.4 | 27.1±5.3 | 0.067 |  |  |  |
| Homocysteine (μM) | 9.8±2.4 | 10.5±2.2 | 0.057 | 11.4±3.3 | 10.7±2.5 | 0.100 |  |  |  |

Supplemental Table 3. Independent factors associated with homocysteine levels in AD patients

| **Variable** | **OR** | ***P*** |
| --- | --- | --- |
| WMH volume | 1.34（1.054, 1.704） | 0.017 |
| Vitamin B12 | 0.996（0.994, 0.998） | 0.000 |
| APOE4 allele | 2.691（1.227, 5.905） | 0.014 |

Supplemental Table 4. Independent factors associated with methylation levels in AD patients

| **Variable** | **OR** | ***P*** |
| --- | --- | --- |
| Vitamin B12 | 1.002（1.001, 1.004） | 0.004 |
| MMSE | 1.155（1.053, 1.268） | 0.002 |
| Age | 0.943（0.898, 0.990） | 0.019 |
